# Determining the Radiation Enhancement Effects of Gold Nanoparticles in Cells in a Combined Treatment with Cisplatin and Radiation at Therapeutic Megavoltage Energies

**DOI:** 10.3390/cancers10050150

**Published:** 2018-05-22

**Authors:** Celina Yang, Kyle Bromma, Wonmo Sung, Jan Schuemann, Devika Chithrani

**Affiliations:** 1 Department of Biomedical Physics, Ryerson University, Toronto, ON M5B 2K3, Canada; celina.yang@ryerson.ca; 2 Department of Physics and Astronomy, University of Victoria, Victoria, BC V8P 5C2, Canada; kbromma@uvic.ca; 3 Department of Radiation Oncology, Massachusetts General Hospital and Harvard Medical School, Boston, MA 02114, USA; WSUNG1@mgh.harvard.edu (W.S.); JSCHUEMANN@mgh.harvard.edu (J.S.)

**Keywords:** gold nanoparticles (GNP), cisplatin, Monte Carlo, radiation, chemoradiation

## Abstract

Combined use of chemotherapy and radiation therapy is commonly used in cancer treatment, but the toxic effects on normal tissue are a major limitation. This study assesses the potential to improve radiation therapy when combining gold nanoparticle (GNP) mediated radiation sensitization with chemoradiation compared to chemoradiation alone. Incorporation of GNPs with 2 Gy, 6 MV (megavoltage) radiation resulted in a 19 ± 6% decrease in survival of MDA-MB-231 cells. Monte-Carlo simulations were performed to assess dosimetric differences in the presence of GNPs in radiation. The results show that physics dosimetry represents a small fraction of the observed effect. The survival fraction of the cells exposed to GNPs, cisplatin, and radiation was 0.16 ± 0.007, while cells treated with cisplatin and radiation only was 0.23 ± 0.011. The presence of GNPs resulted in a 30 ± 6% decrease in the survival, having an additive effect. The concentration of the GNPs and free drug used for this study was 0.3 and 435 nM, respectively. These concentrations are relatively lower and achievable in an in vivo setting. Hence, the results of our study would accelerate the incorporation of GNP-mediated chemoradiation into current cancer therapeutic protocols in the near future.

## 1. Introduction

The integration of chemotherapy with local modalities of radiation therapy is a reasonable approach that has greatly improved the cure rates of solid tumors [1]. However, the major limitation of combining chemotherapy and radiation therapy (chemoradiation) is the normal-tissue toxicity. A radiosensitizer may not have a direct anti-cancer effect or it may be one variety of anticancer drugs that exhibits anti-tumor effects in addition to radiosensitization [2]. Cis-diamminedichloroplatinum (II) or cisplatin (cis-[PtCl_2_(NH_3_)_2_]), which is selected for this study, is considered an effective chemotherapeutic agent used to treat a variety of solid human tumors that exhibits anti-tumor effects in addition to radiosensitization [3,4]. Previous reports suggest that cisplatin entry into cells is not protein-mediated [5]. Eljack et al. showed that cisplatin is capable of passive diffusion across the lipid bilayer membrane [3]. Although there is still a possibility of cisplatin uptake being facilitated, passive diffusion is considered a major uptake pathway of cisplatin into cells [3]. The structure of cisplatin (cis-[PtCl_2_(NH_3_)_2_]) has no net charge and is stable in a high chloride concentration (>100 mM) such as the extracellular matrix [3]. The small neutral compound is capable of diffusing across the cell membrane, and once the compound enters the cytoplasm, the chloride ions dissociate from the platinum ion due to the decrease in chloride concentration in the medium (approximately 4 mM) [3]. The dissociation results in positively charged complexes, [PtCl_2_(NH_3_)_2_ (OH_2_)]^+^ and [PtCl_2_(NH_3_)_2_ (OH_2_)_2_]^2+^ which have a lower rate of permeation than the neutral cisplatin [3,6]. The hydrolyzed product is a potent electrophile that reacts with any nucleophile, including nitrogen donor atoms on nucleic acids [6]. Cisplatin binds to the N7 on purine and causes deoxyribonucleic acid (DNA) damage in cancer cells by forming 1,2- or 1,3-intra-strand or inter-strand crosslinks [7]. The crosslinks alter the structure of the DNA and can result in apoptotic cell death [6,7].

Nanoparticles (NPs) are sub-micrometer sized particles, often smaller than 200 nm which exhibit unique physical and chemical properties [8,9,10]. Among the various NP systems, gold nanoparticles (GNPs) have been used extensively in the field of nanomedicine and cancer research for imaging and therapeutic purposes [11,12,13,14]. GNPs are synthesized in various sizes and shapes and the surface properties can be modified with various small molecules which increases the application possibilities of GNPs [12,13,15,16,17,18]. GNPs are also being used as a radiation dose enhancer in radiation therapy. The use of high-Z elements to improve radiation therapy outcomes has greatly increased in the last decade, with a particular interest in GNPs. Early work by Hainfeld et al. showed their potential as radiosensitizers by demonstrating natural tumor specificity and substantial improvements in tumor control in mice receiving kilovoltage radiotherapy minutes after intravenous injection of GNPs [19]. These results prompted a series of theoretical and experimental studies on the radiosensitizing potential of GNPs. The extent of sensitization with GNPs depends on several factors including the beam energy, size of the NPs, and the radiation dose. Greater radiation sensitization was seen for cells irradiated with lower energy beams (kV) than with higher energy (MV) [15,20]. Schuemann and colleagues [21,22] have performed several Monte-Carlo (MC) studies to calculate the amount of dose enhancement induced by GNPs for multiple irradiation modalities, including kV and MV range photon irradiations. Due to the high cross-section of the photoelectric effect in gold, photons of lower energy have been shown to be more effective for GNP enhanced radiation therapy than those of higher energy. However, as shown in previous studies, GNPs enhance radiation doses in both the kV and MV range in vitro and in vivo [15,23,24]. MV photons are used more commonly in radiation therapy since they can reach tumors located deep within the patient. Hence, in this study, clinically relevant MV energy is selected for GNP-mediated radiation therapy and the dose enhancement using MC simulations based on the experimental parameters used in the study are evaluated.

In this study, peptide-modified GNPs are used in combination with cisplatin—a chemotherapeutic agent that also acts as a radiosensitizer—and radiation to demonstrate that the presence of relatively low concentrations of peptide-modified GNPs improves the therapeutic outcome compared to the same dosages of cisplatin and radiation in MDA-MB-231 cells. To the current authors’ knowledge, this is the first study to demonstrate the effect of GNPs in combination with cisplatin and radiation at a sub-nanomolar concentration using MV range radiation. These novel treatment options may lead to reduced side effects, improving the quality of life of cancer patients in the future.

## 2. Results

### 2.1. Characterization of GNP Constructs

**Figure 1:**
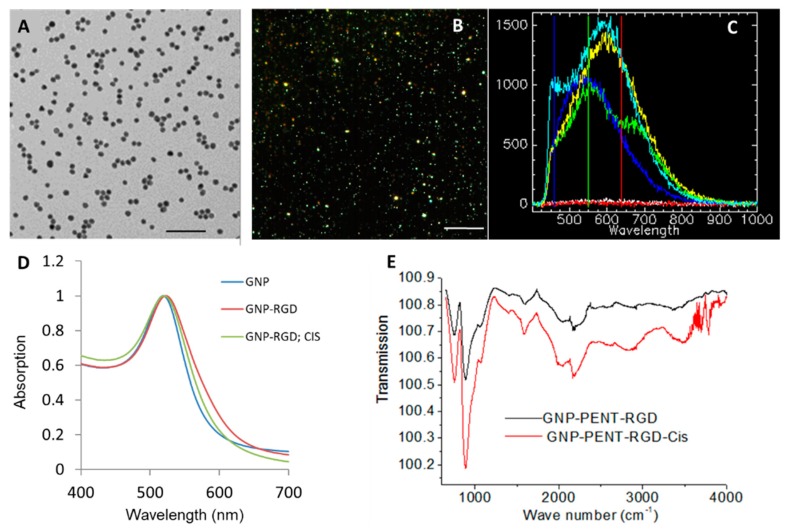
Characterization of gold nanoparticles (GNPs) used in this study (**A**) Transmission Electron Microscopy (TEM) of GNPs used in this study. Scale bar = 100 nm; (**B**) Hyperspectral image of GNPs. Scale bar = 20 µm (**C**) Few spectra of the GNPs from (**B**) and background spectra; (**D**) Ultra-Violet Visible (UV-Vis) spectra of GNP constructs; (**E**) Fourier Transform InfraRed (FTIR) of GNP constructs.

The shape and size of GNPs used for this study is determined with transmission electron microscopy (TEM) as shown in Figure 1A. The approximate core diameter of these GNPs was 10.04 ± 0.89 nm where data are given as means ± S.D. with *n* = 50 NP measurements. Dark field images and a few reflectance spectra from individual GNPs and the background are shown in Figure 1B,C, respectively. The bright dot-like structures are GNP clusters that are imaged without any optical probes. This is possible as GNPs have higher scattering cross sections than cell structures in visible light. The hyperspectral imaging (HSI) technology was used to obtain the reflectance spectra corresponding to each pixel from the dark field images. The spectra taken from the background were flat while spectra taken from GNP clusters had an intensity peaking at a wavelength of 550–650 nm.

The shape of the Ultraviolet-Visible (UV-Vis) spectra shown in Figure 1D did not significantly differ for unmodified GNPs (GNP), peptide modified GNPs (GNP-RGD), and peptide modified GNPs with cisplatin (GNP-RGD; CIS) indicating that cisplatin did not bind to GNPs. Our Fourier Transform InfraRed (FTIR) data in Figure 1E showed that addition of cisplatin into GNP-RGD complex didn’t change the GNP surface. We have performed dynamic light scattering and zeta poetical measurements to further verify that addition of cisplatin to GNP-RGD complex didn’t change the hydrodynamic diameter or the surface charge of the GNP-RGD complex (see Supplementary Material Table S1). 

### 2.2. Cellular Accumulation of GNP Complexes

**Figure 2:**
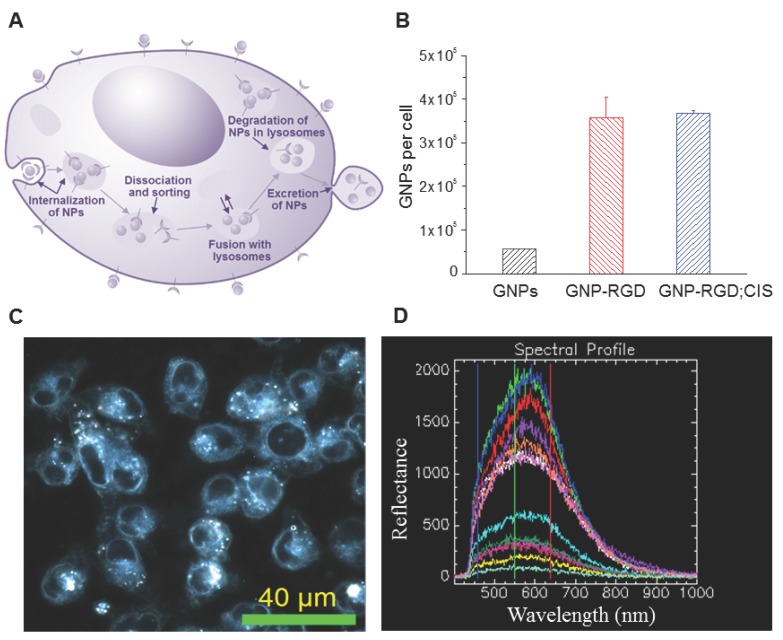
(**A**) Cell uptake pathway of GNPs; (**B**) Accumulation of GNP constructs; (**C**) Dark field image of cells incubated with GNP constructs. Scale bar = 40 µm; (**D**) Few spectra of GNP constructs from (**C**).

Gold nanoparticles were transported through the cell via endo-lyso path as illustrated in Figure 2A. The amount of GNP, GNP-RGD, and GNP-RGD; CIS accumulated with the cells were measured and calculated after 16-h incubation into MDA-MB-231 cells. The concentration of cisplatin used was 435 nM throughout the study, which was the maximum amount that could be added without aggregation of the GNP complex. The number of GNPs accumulated per cell for GNP, GNP-RGD and GNP-RGD; CIS were 56,000 ± 1200, 358,000 ± 47,000, and 367,000 ± 6600, respectively, which is shown in Figure 2B. Modifying the GNP surface with RGD peptides improved the accumulation of the GNPs by 6–7 fold. Addition of cisplatin to the GNP-RGD solution did not significantly change the accumulation of GNP constructs in cells. In order to visualize the internalization of GNP-complexes within cells, the dark field imaging technique was used as shown in Figure 2C. Hyperspectral imaging results in Figure 2D show bright structures within cells are the GNP-complexes localized within vesicles of cells. 

### 2.3. Chemotherapy with Cisplatin in the Presence and Absence of GNPs

**Figure 3:**
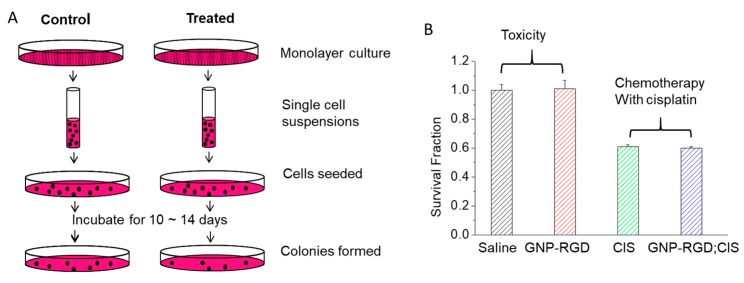
(**A**) Steps used in the clonogenic assay; (**B**) Toxicity and effect of cisplatin action in the absence and presence of GNPs. Data are means ± S.D. for *n* = 9 cell preparations over three independent experimental set-ups.

Toxicity due to GNPs at the concentration used for the study was tested with clonogenic assays. A concentration of 0.3 nM of 10 nm-sized peptide-modified GNPs was used throughout this study. The MDA-MB-231 cells that were incubated with 0.3 nM of 10 nm-sized peptide-modified GNPs (GNP-RGD) did not demonstrate a decrease in survival fraction (SF) of MDA-MB-231 cells compared to the control group that were incubated with the same volume of phosphate buffed saline (PBS). The calculated SF from clonogenic assay is given in Supplementary Materials Section S2. We used a clonogenic assay to test the toxicity since the assay is an accurate measure of cell proliferation over a longer period. The steps taken in this assay are illustrated in Figure 3A. Based on the clonogenic assay results, the concentration of 0.3 nM of 10 nm-sized peptide-modified GNPs that was used in this study had no significant toxic effect on MDA-MB-231 cells when incubated for up to 16 h (*p* > 0.05) (left data panel of Figure 3B). 

The effect of chemotherapy treatment with cisplatin was tested in the absence and presence of GNPs. The effect of GNP-RGD presence in cisplatin treatment was also assessed with clonogenic assays. The MDA-MB-231 cells incubated with GNP-RGD; CIS had no statistically significant change in SF compared to cells incubated with the same concentration of free cisplatin (CIS), with SF of 0.61 ± 0.005 and 0.60 ± 0.005 (right panel of Figure 3B), respectively (*p* > 0.05).

### 2.4. Gold Nanoparticles as a Radiosensitizer in Radiation Therapy

**Figure 4:**
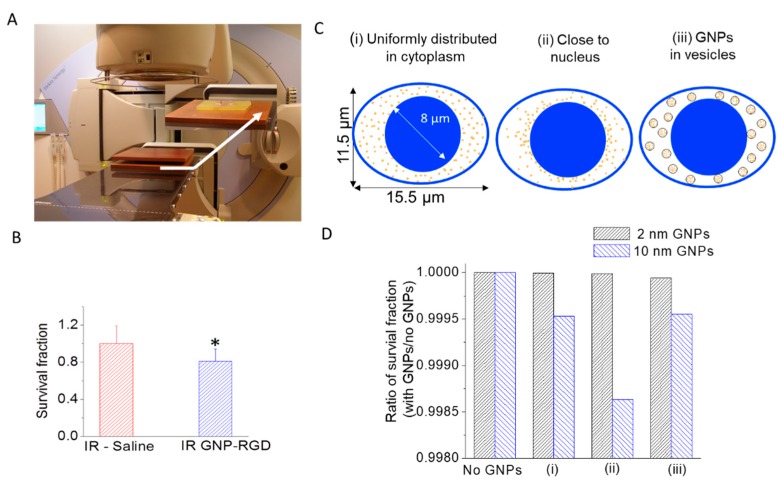
(**A**) Setup of irradiation experiment; (**B**) Cell survival fraction of MDA-MB-231 cells treated with saline (control) and GNP-RGD) (Gold nanoparticles modified with a peptide containing Arg-Gly-Asp tripeptide sequence (RGD)) prior to 2 Gy, 6 MV X-ray radiation. Data are means ± S.E.M. for *n* = 3. * represents statistically significant difference (unpaired *t*-test, *p* < 0.05); (**C**) Schematic of cell geometry and GNP distributions used in the Monte Carlo simulation based effect modeling; (**D**) The estimated reduction in survival fraction when using 2 nm and 10 nm GNPs with 367,000 GNPs internalized in the cell.

Clonogenic assays and immunofluorescence assays were used to assess the therapeutic efficacy of GNP-mediated radiation therapy. Cells internalized with GNP complexes were given a 2 Gy radiation dose using a 6 MV radiation beam as illustrated in Figure 4A. The cells that were irradiated with modified GNPs had a 19 ± 6% (*p* < 0.05) decrease in survival compared to the irradiated control group with a survival fraction of 0.32 ± 0.06 and 0.26 ± 0.03, respectively (Figure 3B). Monte Carlo (MC) simulations were performed to estimate the reduction in cell survival as a function of size and distribution of GNPs (Figure 4C,D). Three cases as shown in Figure 4C are considered: (i) GNPs uniformly distributed in the cytoplasm, (ii) GNPs close to the nucleus, (iii) GNPs located within vesicles. The survival fraction with GNPs was slightly reduced when GNPs were simulated to be within the cytoplasm preferentially in proximity to the nucleus by up to 0.14% for GNPs of 10 nm diameter (Figure 4D ii). The survival fraction was only reduced by 0.04% for the other two scenarios with 10 nm GNPs uniformly distributed either individually or constrained within the vesicles, (Figure 4D i,iii). 2 nm GNPs as frequently used in experiments showed no significant sensitization (less than 0.01%) when the cells internalize the same number of GNPs.

**Figure 5:**
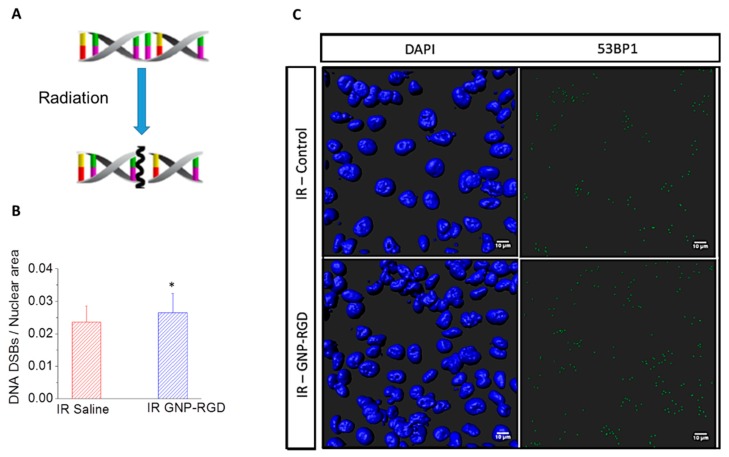
(**A**) Illustration of DNA double strand break from radiation treatment; (**B**) DNA DSBs per nuclear area for MDA-MB-231 cells treated with saline (control) and GNP-RGD prior to 2 Gy, 6 MV radiation; * represents statistically significant difference (unpaired *t*-test, *p* < 0.05) (**C**) Qualitative representation of (**B**). The nucleus is stained with DAPI (shown in blue) and the markers for DNA DSBs, 53BP1, are shown in green. Scale bar = 10 µm.

The amount of damage to cells was also probed using an immunofluorescence assay as shown in Figure 5. The cells treated with GNP-RGD prior to the 2 Gy, 6 MV radiation had a significant increase in DNA Double strand breaks (DSBs) (53BP1 Ser 1778 foci) per nuclear area compared to the control cells (with no GNPs) prior to radiation (Figure 5B,C). The nuclei were stained with DAPI, which is shown in blue, and 53BP1 foci were tagged with Alexa 488, which is shown in green (Figure 5C). The qualitative images were produced by 3D reconstruction of the DAPI, overlayed with green pixels. The quantitative data in Figure 4B was produced by counting the 53BP1 and dividing by the 2D projected area of all the nuclei imaged (*n* = 274 for control; *n* = 310 for GNP-RGD). The results from the clonogenic and immunofluorescence assays confirmed the GNP-mediated radiosensitization.

### 2.5. Combination of Gold Nanoparticles, Cisplatin and Radiation

**Figure 6:**
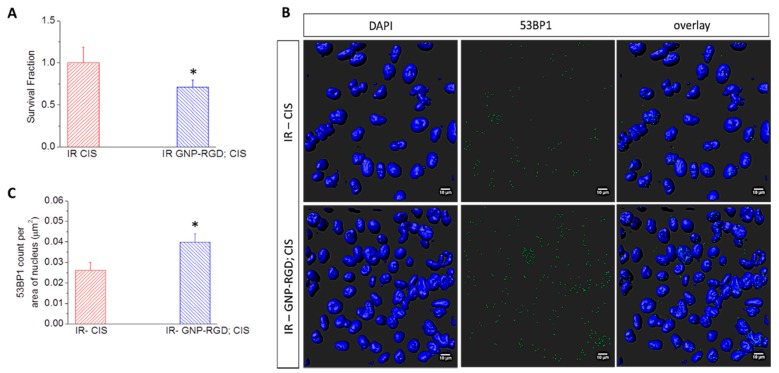
(**A**) Cell survival fraction of MDA-MB-231 cells treated with CIS and GNP-RGD; CIS prior to 2 Gy, 6 MV radiation. Data are means ± S.E.M. for *n* = 3. * represents statistically significant difference (unpaired *t*-test, *p* < 0.05); (**B**) Qualitative representation of DNA DSBs in MDA-MB-231 cells treated with CIS and GNP-RGD; CIS prior to 2 GY, 6 MV X-ray radiation. The nucleus is stained with DAPI (shown in blue) and the markers for DNA DSBs, 53BP1, are shown in green. Scale bar = 10 µm; (**C**) Quantitative analysis of (**B**).

Clonogenic assay and immunofluorescence assays were also used to assess the therapeutic efficacy of GNP-mediated chemoradiation (Figure 6). Different batches of cells were first treated with CIS and GNP-RGD; CIS. After a 16 h incubation period, cells were given a 2 Gy radiation dose using a 6 MV radiation beam. The survival fractions of cells were calculated after the treatment using clonogenic assays and are shown in Figure 6A. The cells treated with GNP-RGD; CIS and radiation (referred to as IR GNP-RGD; CIS) had a 30 ± 6% (*p* < 0.05) decrease in cell survival compared to the cells treated with cisplatin and radiation (referred to as IR CIS). The survival fractions for each treatment option is tabulated in Supplementary Material Table S2 and the sequence of chemoradiation protocol is illustrated in Supplementary Material Table S3. 

The DNA damage was probed after 24 h of the treatment using immunofluorescence assay. The cells were fluorescently tagged with 53BP1 antibodies for probing DNA DSBs. The fixed cells were then imaged with a confocal microscope. As shown in Figure 6B, the qualitative images were produced by 3D reconstruction of the DAPI stained nucleus (shown in blue is DAPI) and overlaying with the 53BP1 (shown in green is the 53BP1). The quantitative data was produced by counting the 53BP1 and divided by the 2D projected area of all the nuclei imaged (*n* = 274 for saline; *n* = 310 for GNP-RGD; *n* = 307 for CIS; *n* = 357 for GNP-RGD; CIS). As shown in Figure 6C, the number of 53BP1 foci per 2D projected z-stacked nuclear area for cells treated with IR—saline, IR GNP-RGD, IR—CIS and IR—GNP-RGD; CIS prior to radiation were 0.024 ± 0.0056, 0.026 ± 0.0059, 0.026 ± 0.0045 and 0.040 ± 0.0044, respectively. These results indicate that the DNA DSBs are increased for cells treated with IR GNP-RGD; CIS compared to all other treatment conditions. The triple combinational treatment of cells treated with GNP-RGD; CIS and radiation had an improved therapeutic result compared to the double combined treatment of cisplatin and radiation.

## 3. Discussion

Combination of radiation with chemotherapy has shown benefits in cancer treatment. The goal of this study was to introduce GNPs into a combined radiation and chemotherapy option. The major internalization pathway of unmodified GNPs in cells is reported to be energy dependent [25,26,27,28,29] with clathrin-mediated endocytosis being the dominant pathway [30]. Once the particles are internalized, an endosomal compartment is formed [31]. The particles are subsequently recycled back to the plasma membrane or progress to lysosomes for degradation [32]. An illustration of the GNP internalization is shown in Figure 2A. The accumulation of GNPs in cells were further improved by modifying their surface with peptide sequences containing the ‘RGD’ amino acid motif. The arginyl-glycyl-aspartic acid (RGD) tripeptide sequence is found in proteins such as Fibronectin, Citronectin, and type I Collagen [33,34]. These three amino acids form the core structure recognized by cell surface receptors and improve the intracellular retention of the NPs [34,35]. The RGD peptide sequence is one of the principle adhesive ligands that are recognized by several integrin receptors [36,37,38]. Improved tumor targeting has been observed in studies using RGD-modified drug constructs because the integrin receptors are overexpressed on tumor cells [39,40,41,42]. Based on previous studies, the 6–7 times improvement in accumulation of RGD modified GNPs found in this study as shown in Figure 2B is reasonable.

New nanoscale systems that are studied to be used with existing treatment modalities should be carefully probed for unwanted attributes. Much experimental work has been done to confirm the non-toxicity of GNPs, but contradictory research results are also present [43]. Several groups studying GNP cytotoxicity concluded that GNP biocompatibility depends on size, surface properties and concentration [43,44,45,46,47,48]. The lack of general consensus on NP toxicity is due to different experimental methods employed, incubation conditions (concentrations and exposure time), variability of sizes and functionalities of GNPs, variability of cell lines, and different measures for toxicity [43,49]. Owing to the numerous parameters that affect toxicity, the assessment of toxicity is rather complicated [50]. In this study, 0.3 nM concentrations of 10 nm GNPs had no signs of toxicity to MDA-MB-231 cells from clonogenic assay results as shown in Figure 3.

The use of chemotherapy treatment in the presence of GNPs was tested before introducing radiation therapy. The initial results in Figure 3B showed that there was no apparent change in the action of the anti-cancer drug, cisplatin, in the presence of GNP constructs. GNP characterization data showed that there were no apparent changes in the size and surface properties of NPs. For example, Ultraviolet-Visible (UV-Vis) spectra showed no aggregation with the addition of cisplatin (Figure 1D). The size range of colloidal GNPs can also be determined by the peak SPR wavelength [51,52]. UV-Vis measurements can also be used to evaluate the functionalization or aggregation of GNPs. When GNPs are successfully functionalized with ligands, the local refractive index at the GNP surface will increase and result in a slight red-shift of the Local Surface Plasmon Resonance (LSPR) while maintaining the overall shape and intensity of the spectra. When GNPs are aggregated from irreversible inter-particle coupling, the LSPR will not only red-shift but the spectra will also broaden. Aggregated GNPs can also be detected visibly by the change in color of the GNP solution from red to blue. The addition of cisplatin to the GNP resulted in no significant broadening of the UV spectra at up to 46 h post formulation as shown in Figure 1D. This signifies that the addition of cisplatin at that concentration does not destabilize the GNP complex. That the addition of cisplatin had no significant effect on the accumulation in cells indicates that the cisplatin has no known interaction with GNP constructs and the uptake pathway of cisplatin molecules does not compete with the uptake of the GNP constructs. The addition of cisplatin did not affect the accumulation of GNP-RGD (Figure 2B), and the presence of GNP-RGD had no effect on the efficacy of the drug, as shown from the clonogenic assay results (Figure 3B). No significant difference in cell survival between cells treated with CIS and GNP-RGD; CIS was found. This indicates that the presence of GNP constructs in the cell does not interfere with the action of the anti-cancer drug.

The local radiation dose can be improved with high atomic number (Z) particles, such as GNPs due to the elevated photoelectric absorption [49,53]. The photoelectric effect, however, predominantly occurs in the kV energy range but kV energy radiation lacks in the ability of deep penetration and therefore it is only used to treat superficial tumors. Therefore, MV energy radiation was used in this study for wider clinical relevance. Clonogenic assays were used to assess the therapeutic efficacy of GNP-mediated radiation therapy. The cells that were irradiated with GNP-RGD had a 19 ± 6% (*p* < 0.05) decrease in survival compared to the irradiated control group (Figure 4B) with survival fractions of 0.25 ± 0.014 and 0.31 ± 0.008 respectively. However, several experimental results have shown dose enhancement with GNPs in the MV range and our results are consistent with previously published studies [15,24,54,55,56,57]. For example, Liu et al. incubated murine cancer cells CT26 with 500 µM of 6.1 nm PEGylated GNPs and observed a dose enhancement of 1.32 with 6 MV X-ray radiation [57]. Jain et al. reported a dose enhancement of 1.29 in MDA-MB-231 cells exposed to 12 µM of 1.9 nm GNPs (Aurovist ^TM^) [24]. Chithrani et al. observed a dose enhancement of 1.17 in HeLa cells exposed to 1 nM of 50 nm citrate-coated (unmodified) GNPs [15]. The decrease in survival fraction by 19 ± 6% for MDA-MB-231 cells exposed 10 nm peptide modified GNPs observed from this study is within the range of enhancement reported of 6 MV GNP sensitization studies mentioned above even with the relatively lower 0.3 nM concentration being used. This enhancement in radiation dose at 6 MV energies is higher than the values predicted through Monte Carlo (MC) calculations shown in Figure 4C. This is likely due to the fact that the MC simulations only calculate effects of physical dose enhancements which are confined in small volumes around the GNPs. In this experiment GNPs do not enter the nucleus, which is assumed to be the target for GNP induced damages. This suggests that other, longer ranged effects may be responsible for the majority of the biologically observed effect. For example, the changes in the chemistry around GNPs have not been included in the simulation. However, MC calculations have been significantly improved over the last decades. We were able to show that cell survival fraction varies depending on the distribution of GNPs within the cells. We used our experimental NP accumulation data for the prediction of survival fraction and the NP concentration used for the study was orders of magnitude lower than previously published data, yet the MC simulations were still able to predict a small GNP radiosensitization effect. This is a clear example of the improvements made to currently used MC models and the models continue to be improved.

**Figure 7:**
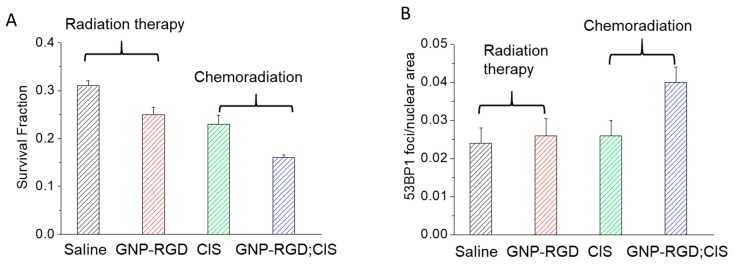
GNP-mediated radiation therapy vs. chemoradiation. (**A**,**B**) Clonogenic and DNA DSBs assay results showing how the presence of GNPs affect the treatment outcome.

The final goal of this study was to investigate the effect of GNP constructs in chemoradiation. Our results show that cells treated with GNP-RGD; CIS and radiation had a 30 ± 6% (*p* < 0.05) decrease in cell survival compared to cells treated with cisplatin and radiation with the survival fraction of 0.16 ± 0.007 and 0.23 ± 0.011, respectively (see Figure 7A). Previous studies have shown that the combined use of chemotherapy and radiation therapy (chemoradiation) has improved the cure rates of solid tumors in patients [1]. The presence of peptide modified GNPs was shown to have an additive effect to cisplatin and radiation using the Bliss Independence Criteria (see the Supplementary Materials Section S4) [58]. Cui et al. also recently reported that the combination of peptide (RME) modified GNPs and 12 µM cisplatin produced additive effects at lower energies (225 kV) in MDA-MB-231 cells [59]. The results of this study indicate that an additive effect can also be achieved with lower concentrations of the constructs and at a higher energy radiation. Higher energy (MV range as opposed to kV range) radiation is the primary radiation modality used in the clinic and is capable of deeper penetration, and therefore, has a wider clinical relevance. 

The outcome of GNP-mediated radiation therapy and chemoradiation was also assessed by mapping the DNA damage as summarized in Figure 7. DNA double-strand breaks (DSBs) are considered the most harmful type of DNA lesions because unrepaired DSBs are sufficient to trigger permanent growth arrest and cell death [60,61,62]. Cells respond to DSBs by mounting a complex signaling network, referred to as the DSB response, that coordinates DNA repair reactions with DNA damage checkpoint activation and chromatin reorganization [63]. An important regulator of DSB signaling is 53BP1 [64]. As an early event in the recognition of the breaks, 53BP1 becomes hyperphosphorylated after radiation and rapidly redistributes into distinct nuclear foci. The average number of 53BP1 foci peaks 30 min post cell exposure to DNA DSB inducing agents and decreases over time with kinetics that parallel the rate of DNA repair over time returning to baseline 16 h post exposure [61,65]. Since the treated cells were fixed 24 h post treatment, the 53BP1 foci detected were an indicator of the DNA DSBs that have a lower probability of being repaired. A single persistent DNA DSB may be sufficient to induce cell death through apoptosis [66,67]. As shown in Figure 7, the number of 53BP1 foci per unit nuclear area were increased when GNPs were introduced into the chemoradiation protocol. To our knowledge, this is the first time that DNA damage was analyzed for GNP-mediated chemoradiation.

The results of our study show that the triple combinational treatment of cells with GNPs, chemotherapeutic drug, and radiation could produce an improved therapeutic result compared to the combined conventional treatment of cisplatin and radiation. In the clinics, multiple dosages of chemotherapy and multiple dosages of radiation are generally prescribed to the patient. The usage of GNPs in combination with chemotherapeutic agents and radiation has shown statistically significant improvement in this study and the effectiveness can become more apparent for multiple treatments [68]. It is generally recognized that in vitro data cannot be extrapolated directly to in vivo or clinical settings since assays in vitro assays do not account for tumor microenvironmental factors and the fact that tumors may contain clonogenic subpopulations of cells with different sensitivity to radiation or chemotherapeutic of interest [68]. However, a decrease in SF with the presence of GNP-RGD for one dose of chemotherapeutics can potentially mean less number of treatments in an overall treatment regimen. The average number of cells surviving for a tumor containing 10^12^ cells and the corresponding tumor control probability have been calculated and shown in Supplementary Materials Sections S5 and S6 for proof of principle purposes, respectively. It clearly shows the fact that a small difference in survival fraction can lead to a significant difference in a clinical setting where multiple dosages are being used. Gold nanoparticles acting as a radiosensitizers could potentially be another addition to the toolbox of combination therapy that some patients could benefit from since the presence of GNPs does not hinder the action of chemotherapeutic drugs.

In this study, a relatively small (sub-nanomolar) concentration of GNPs was used to observe the therapeutic enhancement. The accumulation of GNPs in multi-layer models or in vivo settings require GNPs to pass through more barriers prior to cell entry, therefore, a higher incubation concentration is required to result in comparative results from in vitro experiments. The authors have also studied the accumulation of GNPs in monolayer in vitro, multilayer models, and in vivo settings [69]. Therefore, improvement in therapeutic results with a relatively low incubation concentration of GNPs in vitro can be seen as a first predicator for successful outcomes in in vivo studies with appropriate modifications.

## 4. Materials and Methods

### 4.1. Preparation of Peptide Modified GNP Constructs

GNPs of size 10 nm were synthesized using the citrate reduction method. 300 μL of 1% chloroauric acid (HAuCl_4_·3H_2_O) (Sigma-Aldrich, St. Louis, MO, USA) was added to 30 mL of double–distilled water and heated on a hot plate while stirring. Once it reached the boiling point, 1 mL of 1% sodium citrate tribasic dehydrate (HOC(COONa)(CH_2_COONa)_2_·2H_2_O) (Sigma-Aldrich) was added to form NPs of diameter 10 nm. After the color of the solution changed from dark blue to bright red, the solution was left to boil for another five minutes while stirring. Finally, the GNP solution was brought to room temperature while stirring. Peptide modified GNP constructs were assembled by first conjugating the GNPs with a CALNN pentapeptide (AnaSpec, San Jose, CA, USA), with approximately 300 peptides per GNP for stabilization purposes. The peptide with the NH_2_-Cys-Lys-Lys-Lys-Lys-Lys-Lys-Gly-Gly-**Arg-Gly-Asp**-Met-Phe-Gly-COOH sequence (AnaSpec, San Jose, USA) was added in a 16 to 20 peptide/GNP ratio. This peptide modified GNP construct will be labelled GNP-RGD. The tripeptide sequence Arg-Gly-Asp is referred to as RGD. Cisplatin (Tocris Bioscience Bristol, UK) was added to the GNP-RGD construct at approximately 620 molecules/GNP. It was expected that cisplatin molecules do not have an interaction with the GNPs and remain in the mixture. This construct will be labelled as GNP-RGD; CIS (CIS refers to cisplatin). We use a semicolon instead of a hyphen to indicate addition of cisplatin to the GNP-RGD solution.

### 4.2. Characterization of NPs

The core size and shape of the GNPs were obtained using transmission electron microscopy (TEM) using Hitachi H7000 TEM (Hitachi Coop., Tokyo, Japan) operated at 100 keV. The core size was measured with ImageJ software (NIH, Bethesda, MD, USA). The UV-Vis spectra were obtained with Lambda 20 (Perkin Elmer, Waltham, MA, USA) to observe the presence of aggregation of the GNP constructs.

### 4.3. Cell Culture and Particle Delivery

Human breast cancer cell line, MDA-MB-231 cells, were used for this study. The cells were cultured in Dulbecco’s Modified Eagle’s Medium (DMEM) supplemented with 10% Fetal Bovine Serum (FBS) at 37 °C humidified incubator with 5% CO_2_. The cells were exposed to either Phosphate-Buffered Saline (PBS), 0.3 nM of GNP-RGD, 435 nM of CIS, or 0.3 nM of GNP-RGD and 435 nM of CIS for sixteen hours prior to radiation and/or clonogenic assays. For optical imaging purposes, the cells were placed on glass coverslips, grown to 75–80% confluency. Following the exposure to the various constructs, the coverslips were washed three times with PBS. Subsequently, the cells were fixed with 4% paraformaldehyde in PBS for 20 min at room temperature and then mounted onto glass slides.

### 4.4. Quantitative Assessment of GNP Accumulation in Cells

GNP accumulation in cells was quantified using inductively coupled plasma atomic emission spectroscopy ((ICP-AES) Optima 7300 DV, Perkin Elmer Inc., Waltham, USA). Following sixteen hours of incubation with GNPs, the cells were washed three times with Phosphate Buffer Saline (PBS) and the cells were suspended from the monolayer cultures with 0.25% trypsin-EDTA (Gibco Invitrogen, Carlsbad, California) for quantification of GNPs present per cell. Cells were counted with either a hemocytometer (Hausser Scientific, Horsham, PA, USA) or a Vi-CELL XR automated cell counter (Beckman Coulter, Brea, CA, USA) and then treated with aqua regia (mixture of 25% hydrochloric acid (HCl) (Sigma-Aldrich) and 75% nitric acid (HNO_3_) (Caledon Laboratories Ltd., Georgetown, Canada) in a ratio of 3:1 *v/v*) in a silica oil bath. The samples were diluted and concentrations of gold (Au) atoms were measured in (mg/L) with the Optima 7300 DV ICP AES (Perkin Elmer, Waltham, MA, USA). The number of GNPs of each sample was calculated.

### 4.5. Clonogenic Cell Survival

After each treatment, the MDA-MB-231 cells were trypsinized to yield single-cell suspensions and counted. The required number of cells for control and treatment samples was calculated, placed on 60 mm tissue culture dishes and evenly distributed on their surfaces. The cells were grown in culture in the 37 °C humidified incubator with 5% CO_2_ for 10–14 days to allow sizeable colonies to form. Once colonies were formed, the dishes were stained and fixed with 0.1% of methylene blue (BioSho, Burlington, Canada) in 70% ethyl alcohol (Fisherbrand, Pittsburgh, USA) for 1 h. The stained dishes were rinsed in tap water and left to air-dry overnight. The air-dried control dishes were then counted to obtain the plating efficiency (PE), where PE = Number of colonies counted/Number of cells plated. The colonies of treatment samples were counted and the survival fractions (SF) were obtained by SF = Number of colonies counted/(Number of cells plated × PE). 

### 4.6. Setup for Radiation Experiments

The cells were grown in six-well tissue culture dishes (6 mm media, 15 mm air) and incubated with GNP constructs 16 h prior to irradiation with a 2 Gy single fraction of 6 MV X-rays with Agility^TM^ Linac (Elekta Oncology Systems, Stockholm, Sweden), at a dose rate of 600 MU/min, and field size of 20 × 20 cm^2^. PMMA bolus and superflab, equal to the depth of the culture dish, were used to surround the culture dish with water equivalent material in lieu of air. Solid water was placed under and above the dish to achieve proper scatter conditions and to set the monolayer source-to-axis distance (SAD) to 100 cm at a depth of 10 cm. Since the culture dishes contain air pockets, the setup was scanned in CT to verify the dose distribution in a treatment planning system. 

### 4.7. Immunofluorescence Assay for Probing DNA Damage

The primary antibody used was rabbit anti-p53 Binding Protein 1 (53BP1) (1:200; Cell Signaling Technology, Danvers, USA). Secondary antibody used was Alexa Fluor488 anti-rabbit IgG (1:500; Life Technologies, Carlsbad, USA). The cells were embedded with VECTASHIELD Mounting Medium containing DAPI (Vector Laboratories, Burlingame, CA, USA). The slides were imaged and analyzed with a LSM 700 confocal microscope (Carl Zeiss Microscopy, Jena, Germany) and the Imaris software (Bitplane, Zurich, Switzerland). The slides were imaged along the z-stack to cover the depth of the nuclei. The quantitative data was produced by counting the 53BP1 and divided by the 2D projected area of all the nuclei imaged. The number of nuclear foci per cell was counted in at least 200 cells.

### 4.8. Immunohistochemistry Monte-Carlo Simulations and Effect Modelling

We performed MC calculations using the quantification data presented in this study. For example, 367 K GNPs were assumed to either be taken up individually or located inside 3670 vesicles with 100 GNPs per vesicle. In the latter case, the GNPs were uniformly distributed inside each vesicle, each with 500 nm diameter. Individual GNPs or vesicle distributions were randomly selected either uniformly within the cytoplasm or using an exponentially decreasing likelihood with distance from the nucleus. A schematic representation of the cell and GNP geometries is shown in Figure 4C. Radial dose distributions were superimposed to calculate radiation dose to the nucleus and predict cell survival fractions. To obtain radial dose distributions around the GNPs, radiation interactions were simulated using TOPAS-nBio (the nanometer biology extension of the Tool for Particle Simulation, TOPAS, version 3.0.1) [70,71], which is layered on top of Geant4 version 10.2.p1 using the Geant4-DNA extension [72,73]. A single, bare (uncoated) GNP was irradiated with particle showers from a 6 MV Varian linear accelerator. The particles incident on the GNPs were acquired at 10 cm depth in a water phantom following the experimental setup. Radial dose distributions around the GNPs were calculated and used in an adaptation of the local effect model (GNP-LEM) [21,22] to calculate survival fractions in the presence of GNPs of 2 nm, and 10 nm diameter. Modeling parameters were selected to represent human breast cancer cells MDA-MB-231. For this study, we used 15.5 μm by 11.5 μm as major and minor axis elliptical cell diameters with an 8 μm diameter nucleus at the center [21,24]. These cells were characterized as having radiation response parameters of α = 0.002, β = 0.079 for 6 MV photons [24]. 

### 4.9. Hyperspectral Imaging

CytoViva technology in combination with dark field microscopy was used to image the GNP distribution within cells. The microscope is a dark-field imaging system that uses oblique angle lighting. This imaging system allows confirmation of GNP spectra despite the interaction of NPs with cells or tissue. NPs appear bright due to high scattering cross-sections of GNPs. To confirm the spectra of GNPs, Spectral Angle Mapping (SAM) was performed. SAM determines the presence of GNPs in the input image by comparing unknown spectra in the acquired hyperspectral image to a user-defined spectrum of GNPs in these experiments. This hyperspectral imaging of GNPs in cells and tissues was practical since it does not require optical labeling of the GNPs. It is also possible to extract spectral information from each pixel for verification purposes.

### 4.10. Statistical Analysis

Data for clonogenic assays are displayed as mean ± standard error with at least three repeats. Statistical analyses were performed using the IBM SPSS Statistics (IBM Corporation, Armonk, NY, USA). A two-sample *t*-test was used to measure statistical significance between pairs of results. For statistical analysis among three or more groups, one-way analysis of variance (ANOVA) was used and subsequent multiple comparisons with Bonferroni correction was performed in any statistical significance was detected by the ANOVA F-test. A *p*-value of less than 0.05 was considered to be significant.

## 5. Conclusions

The results of this work demonstrate that using peptide modified GNPs in combined chemotherapy (435 nM cisplatin) and radiation therapy (single fraction of 2 Gy of 6 MV X-ray) significantly enhanced therapeutic results by 30 ± 6%. The results are summarized in Figure 7. The triple combined effect of GNP-RGD; CIS and radiation both indicated an additive effect examined with the Bliss Independence Criteria. This signifies that the GNP platform can be utilized in combined chemotherapy and radiation therapy with chemotherapeutic agents that do not conjugate onto the surface of the GNPs. The results also show that the incubation of RGD modified GNPs at a relatively low concentration (0.3 nM) can improve combined chemotherapy and radiation even at a MV energy radiation. Clinically, multiple dosages of chemotherapy and radiation are generally prescribed. The GNP platform that can be used with conjugated and unconjugated chemotherapeutic agent along with radiation will be beneficial in treatment plans that involve multiple dosages of various chemotherapeutics and multiple fractions of radiation. Further modifications to this GNP-based platform will have to be performed and tested in future in vivo studies.

## Supplementary Materials

The following are available online at http://www.mdpi.com/2072-6694/10/5/150/s1.
