# Supplementary material for: Determining the Radiation Enhancement Effects of Gold Nanoparticles in Cells in a Combined Treatment with Cisplatin and Radiation at Therapeutic Megavoltage Energies
Source: Cancers (Basel). 2018 May 22;10(5):150. doi: 10.3390/cancers10050150 (PMC5977123; doi:10.3390/cancers10050150)
Supplement: Supplementary file 1 [file cancers-10-00150-s001.pdf]

Determining the radiation enhancement effects of gold nanoparticles in cells in a combined treatment with Cisplatin and radiation at therapeutic megavoltage energies

Supplementary section S1

Table S1. Characterization of nanoparticles using Dynamic Light Scattering (DLS) and Zeta potential measurements

	Hydrodynamic diameter (nm)	Zeta potential (meV)
GNP	18.5 ± 0.2	-28.1 ± 1.2
GNP-RGD	19.2 ± 0.1	-12.1 ± 1.7
GNP-RGD; CIS	19.3 ± 0.2	-12.2 ± 1.3

Supplementary section S2

Table S2. Summary of survival fractions for each treatment option

Treatment condition		Mean	Standard Error of Mean
Non-Irradiated	Saline (control)	1	0.043
	GNP-RGD	1.01	0.060
	CIS	0.61	0.005
	GNP-RGD; CIS	0.60	0.005
Irradiated	Saline (control)	0.31	0.008
	GNP-RGD	0.25	0.014
	CIS	0.23	0.011
	GNP-RGD; CIS	0.16	0.007

Supplementary section S3

Combination therapy protocol and experimental set up used for the study

Table S3. Sequence of the chemoradiation protocol used for the radiation experiments.

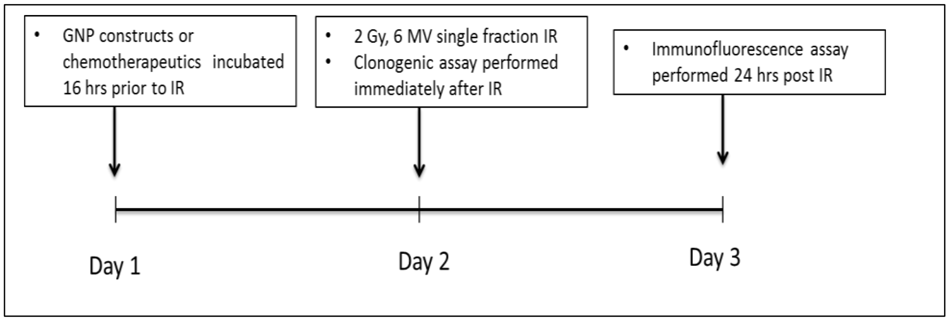

Supplementary section S4

Bliss Independence Criterion

One of the most commonly used models to study combined effects of substances *in vivo* and *in vitro* is the Bliss Independence Criterion as reference [1, 2]. The Bliss criterion for two toxic agents to have an additive effect is expressed by the following equation:

$$E(x,y) = E(x) + E(y) - E(x) * E(y) \qquad (1)$$

where E is the fractional effect (between 0 and 1), x and y are the doses of two compounds in a combinational experiment. If the experimental effect is larger than the calculated value, the experimental result indicates a synergistic effect. If the experimental effect is smaller than the calculated value, the experimental result indicates an antagonistic effect. Otherwise, the effect is additive [1-3]. The Bliss equations are applicable to experimental data for single points and entire dose-response curves but the main assumption of the Bliss Independence Criterion is that the toxic agents act independently from one another [1, 2]. The Bliss Independence Criteria have been used to analyze synergism between multiple modes of treatments.

<Table S4> Comparison of predicted effect using the Bliss Independence Criteria with experimental values.

	Calculated effect from Eq (1)	Experimental Effect (1-SF)	Difference between calculated and experimental
IR GNP-RGD; CIS	0.82 ± 0.02	0.84 ± 0.007	0.02 ± 0.02

The expected additive effect of IR GNP-RGD; CIS was calculated to be 0.82 ± 0.02 which also is within the range of the experimental effect of 0.84 ± 0.007. Since the difference of zero is within the propagated uncertainty range of the difference for both IR GNP-RGD-BLM and IR GNP-RGD; CIS, the calculated effect and the experimental effect can be concluded to agree. The triple combined effect of GNP-RGD; CIS and radiation both indicate an additive effect with the assumption that the chemotherapeutic (GNP-RGD; CIS) and the physical agent (radiation) is independent.

Supplementary section S5

Clinical relevance of the in vitro results

In the clinics, multiple dosages of chemotherapy and multiple dosages of radiation are generally prescribed to the patient and the schedule, dosage of treatment is different from patient to patient. The usage of GNP-

RGD in combination with chemotherapeutic agents, radiation, and combined chemotherapeutic agents with radiation has shown statistically significant improvement in this study and the effectiveness (X) can become more apparent for multiple treatments. Assuming that each treatment is equally effective, and there is no cell proliferation between treatments, the survival following n treatments is given by X^n, which is a concept introduced by Hill and Bristow [4]. In this case, X is the survival fraction (SF). The survival fraction post 10, and 20 treatments of the various permutations of GNP-RGD, chemo (CIS), and radiation and the percentage decrease of the condition pairs are summarized in Table S4.

Table S5. Values of Survival Fraction and Percentage Difference of Various Treatments

Treatment condition		Experimental SF	% decrease	Predicted SF post 10 treatment	% decrease	Predicted SF post 20 treatment	% decrease
Radiation therapy (RT)	Saline (control)	0.31	19	$8x10^{-6}$	88	$7x10^{-11}$	99
GNP-mediated RT	GNP-RGD	0.25		$1x10^{-6}$		$9x10^{-13}$	
Chemoradiation	CIS	0.23	30	$4x10^{-7}$	98	$4x10^{-15}$	99.9
GNP-mediated chemoradiation	GNP-RGD; CIS	0.16		$1x10^{-8}$		$2x10^{-18}$	

As shown in Table S4, small differences in survival can translate into large differences and therefore a larger significance during a course of multiple treatments [4].

<u>Supplementary section S6</u>

<u>Tumor control probability</u>

The probability of tumour control can be estimated from the following equation:

$$P_{0=e^{-a}}$$

Where P_0 is the probability that a tumour will contain no survival stem cells, a is the average number of cells surviving [4]. To achieve tumour control, all tumour stem cells must be killed [4]. Tumour stem cells are

referred to the limited proportion of tumour cells with the capacity for cell proliferation [5]. Cells from human tumours have been found to be able to generate colonies in adequate nutrient environment, however, the proportion of cells that generate colonies have been found to be low (less than 1 percent) which suggests a low proportion of tumour stem cells [5].

A small difference in survival can translate into large differences over multiple treatments and that can also lead to a significant difference in tumour control probability. An example to show the difference in tumour control probability is shown in Table S5.

Table S5. Comparison of tumour control probability extended from experimental SF values

Treatment condition		Experimental SF	SF post 20 treatment	Average number of cells surviving for a tumour containing 10^{12} cells	Tumour control probability
GNP-mediated RT	GNP-RGD	0.25	$9x10^{-13}$	0.9	0.4
GNP-mediated chemoradiation	GNP-RGD: CIS	0.16	$2x10^{-18}$	0.00002	0.999

References:

1. Goldoni, M. J., Carolina. *Toxicology in vitro* **2007,** 21.5, 759-769.

2. Bliss, C. *Annals of applied biology* **1939,** 26, (3), 585-615.

3. Lee, J. J. K., M.; Ayers, G.D.; Lotan, R. *Journal of Biopharmaceutical Statistics* **2007,** 17, 461+480.

4. Hill, R. P. B., Robert B., The scientific basis of radiotherapy. In *The basic science of oncology*, Tannock, I. F. H., Richard P.; Bristrow, Robert G.; Harrington, Lea, Ed. McGraw-Hill: Toronto, 2008; pp 289-321.

5. Donovan, J. C. H. S., Joyce; Tannock, Ian F., Cell proliferation and tumor growth. In *The basic science of oncology*, 4 ed.; Tannock, I. F. H., Richard P.; Bristrow, Robert G.; Harrington, Lea, Ed. McGraw-Hill: Toronto, 2005; pp 167-193.
